# Deploying a rapid point of care polymerase chain reaction test for SARS-CoV-2 in a clinical research unit to ensure healthy volunteer safety

**DOI:** 10.4155/bio-2021-0079

**Published:** 2021-09-13

**Authors:** Radboud van Trigt, Jason Neat, Jan Leendert Brouwer, Amanda Hays, Hans Westerhof

**Affiliations:** ^1^ICON Plc, Amerikaweg 18, Assen 9403TK, The Netherlands; ^2^Jason Neat: ICON Plc, 10836 Strang Line Road, Lenexa, KS 66215, USA; ^3^ICON Plc, Van Swietenlaan 6, Groningen, 9728NZ, The Netherlands; ^4^BioAgilytix, 2300 Englert Drive, Durham, NC 27713, USA

**Keywords:** COVID-19, CRU, Phase I clinical trials, point-of-care PCR, SARS-CoV-2 testing

## Abstract

The entire world was severely affected by the outbreak of the SARS-CoV-2 virus. Early phase clinical research was no exception and clinical healthy volunteer trials were halted across the globe. To enable continuation of development of new drugs, we developed a testing strategy for nonsymptomatic trial participants in an early stage of the outbreak. A point-of-care polymerase chain reaction test combined with a gold standard polymerase chain reaction test and strict social distancing and hygiene measures limited the number of infected subjects entering our clinical research units and reduced further spread for the duration of the clinical trial. Thus, proving efficacy of this strategy to allow safe and effective continuation of early phase clinical trials during the COVID-19 pandemic.

The outbreak of the COVID-19 pandemic in 2020 has led to an unparalleled disruption of society, including a slowdown or in some cases, halt, in many clinical research activities for the development of new drugs. Clinical research units (CRUs) conducting Phase I trials were affected on a global scale. PRA Health Sciences (now ICON plc) site located in Groningen (The Netherlands) had to cease studies in May 2020 due to prioritization of back up intensive care beds that could no longer be guaranteed by local hospitals, whereas in ICON’s US-based CRUs in Lenexa (KS) and Salt Lake City (UT), operations continued but were drastically reduced. This reduction in the US CRUs productivity was due to increased quarantine measures driven by local, state and national recommendations and by sponsor selective trial delays or pauses due to risk driven circumstance around the therapy, volunteers and COVID-19. Given the criticality of current therapies in development and the necessary development of COVID-19 therapies, ICON recognized the need to rapidly change it’s processes in response to the evolving pandemic and enable continuity in clinical trials while maintaining healthy volunteer safety and safety of contributing CRU staff alike. In addition and recognizing the gravity of the novel pandemic on drug development and subject safety, the US FDA had also created guidance on the conduct of clinical trials during the COVID-19 public health emergency [1]. As such, clinical and bioanalytical scientists at ICON collaborated in order to find safe and realistic ways to restart activities for drug development. In this article, we review the strategies, process, systems, tests and results that were deployed and achieved by ICON in order to facilitate continuity in our US and European (EU) CRUs.

During the early days of the pandemic, and the subsequent months to follow, there were many unknowns surrounding the SARS-CoV-2 virus including lack of knowledge on appropriate testing procedures, relevant acceptance criteria, systems and continued stable supply of assay reagents. Hundreds of assays to detect the virus were under development and in the US, through the FDA, Emergency Use Authorizations were requested and in many cases granted as noted on the FDA website [2]. For EU, these were approved through Conformité Européene mark. Approximately half of these new assays for SARS-CoV-2 were detecting viral RNA using polymerase chain reaction (PCR) methodology from nasal, nasopharyngeal or oropharyngeal swab samples [3]. Availability of reagents and equipment was often limited, and some suppliers could not guarantee consistent quality or timely supply.

Central laboratories that were offering PCR-based tests at the time were mainly running samples from hospitalized patients in batches and had limited capacity given the throughput challenges they were facing. In addition, in the early days of the pandemic, and due to limited supplies, testing authorization was only made available for symptomatic cases and by physician request. Hence, turnaround time for samples taken from healthy volunteers were at least 24 h from sample collection to getting results back and sometimes even as long as 96 h. This implied that either testing through this route was not possible, or that all healthy volunteers would need to be quarantined for the entire period of time until results before admitted to the CRU. Although we considered this, it was not an efficient nor practical procedure for our CRUs.

Healthy volunteers participating in clinical trials often spend multiple days in the CRU; and thus, they have to be tested before entering the unit in order to mitigate potential exposure to the virus. In addition to assay and results availability limitations, access and authorization of use in our clinical setting, it was considered of utmost importance to implement SARS-CoV-2 assays with sufficient sensitivity in order to avoid false negative results. Ideally, we were also looking for rapid (30 min or less) reporting requiring onsite analysis.

It was under these circumstances and conditions that we selected commercial PCR-based point-of-care (PoC) assays for testing all healthy volunteers before entering the CRUs. These instruments rapidly determine viral RNA in a buffer from nasal, nasopharyngeal or oropharyngeal swabs, but only for a single individual measurement. A cartridge contains all necessary reagents for sample analysis and results are read from the screen after 20 min. A control RNA amplification is used to determine the validity of the result and; thus, the quality of the swab sample collected. The cycle threshold (Ct) value is read from the screen and the software determines the result as positive, negative or undetermined. Due to limited global availability and regulatory acceptance in the EU and the US, we had to implement systems from different vendors per region; however, deploying them with similar principles. Multiple instruments of the same type were purchased in order to ensure sufficient throughput and were based on average required check-in volumes on a given day.

Healthy study participants went through initial screening procedures specific for the clinical trial they were entering to assess their eligibility to participate. Once qualified, at check-in at the CRU they were asked a series of questions (SARS-CoV-2 exposure related) and were temperature checked. They were than screened for SARS-CoV-2 by the respective PoC assay. A negative result allowed admittance into the study and the CRU. In addition, given many positive patients will not test positive for 2–5 days after exposure [4], it is necessary to test a second time a few days later to confirm the initial test result. Within the CRUs regular health checks including temperature measurement were performed. Additionally, social distancing measures, personal protection using masks and hand sanitizing were practiced reducing the risk of contracting the virus or spreading. For clinical research in healthy volunteers, it is also important to have check-in results available in a timely fashion. In the case of a positive subject, we were depending on a swift source and contact investigation to further mitigate infectious risks within the clinic. This task was performed by a core COVID-19 team, consisting of upper management, nurses and local doctors that were available 24/7.

## Materials & methods

In the CRU in the Netherlands, VitaPCR™ RT-PCR instruments (Credo Diagnostics Biomedical, Singapore) running the SARS-CoV-2 assay were implemented. This is a rapid molecular *in vitro* diagnostic test utilizing a real-time reverse transcription-PCR (RT-PCR) amplification technology for the qualitative detection of SARS-CoV-2. The assay measures 3 genes. The first is a *nucleocapsid N-encoding* gene, the second is a conserved sequence from SARS-like viruses and the third gene is a non-virus related human *β-globin* gene to monitor the cellular content as a surrogate marker of the specimen quality [5]. The assay was used according to the manufacturer’s instructions with daily checks for each instrument. Before analyzing human samples, the assay was implemented in the CRU's clinical chemistry laboratory and its performance evaluated by repeatedly measuring positive and negative controls from the assay, but also samples from external sources.

The analytical sensitivity of the assay, defined as the LOD, is 2.73 copies per μl as reported by the manufacturer. We confirmed this sensitivity using an externally verified control sample. Later, the suitability of using this test system for clinical practice in a hospital setting was described [6].

In the CRUs in the USA, the Accula SARS-Cov-2 Test on the Accula Dock instrument (Mesa Biotech Inc., CA, USA) was implemented. This test has the same principle as the VitaPCR RT-PCR assay; a test cassette contains internal process positive and negative controls, enzymes, reagents and a test strip. The Accula Dock instruments controls the reaction conditions and fluid movements in the test cassette. The analyte is the N-gene of the SARS-CoV-2 viral RNA. The LOD was reported by the manufacturer as 200 copies per reaction of 5 μl, or 40 copies per μl. The testing system has received an Emergency Use Authorization status in the USA for patient care settings (ref to FDA website; see references). Authorization of use in the respective CRU was confirmed by Emergency Use Authorizations and local state Clinical Laboratory Improvement Amendments (CLIA) authorities.

As the sample quality of the swabs can be limiting for the reliability of the result [7], significant attention was given to training the clinical staff for collecting swabs according to strict and consistent procedures. A standard operating procedure describes the procedure for collection of the swabs. The training of a healthcare workers (physician or a research nurse) was performed by a restricted set of trainers. After the training, a practical test was performed before the healthcare worker is allowed to perform swabs on clinical trial participants.

It was known that shortly after SARS-CoV-2 infection, most patients would have no detectable viral RNA due to a very low viral load [3]. In order to deal with that issue and the slightly reduced sensitivity of the PoC test, we elected to repeat the swab procedure on day 2 of the volunteer’s stay in the CRU. The second swab samples were tested by a commercial local laboratory using their routine PCR procedures. For clinical studies that administered (potentially) immune suppressant compounds, we also elected to perform additional sampling on multiple days to further reduce the risk for the clinical trial subjects. In addition, the PoC PCR test was used for testing CRU staff in cases where SARS-CoV-2 infections were suspected based on symptoms or after possible exposure.

## Results

ICON had performed 5675 tests in the Netherlands CRU using the VitaPCR RT-PCR test until 12 April 2021. From these, 5597 gave a negative result (98.6%). In total 40 samples were found positive for SARS-CoV-2 (0.70%). However, from the total of 40 positive results, 18 were from staff members who were tested because of COVID-19 related symptoms or suspected exposure. In total 969 tests were performed on staff members, so 1.86% of the total samples from staff members tested positive. Similarly, the positivity rate in the healthy volunteer population was 0.47% (22 out of 4706). From these 22 positive samples, 11 were collected at check-in, so before the subject entered the trial or the CRU, the remaining 11 positive results were obtained from tests performed during the residential period, often as a result from a suspected exposure or to confirm an external positive test result. In total 38 samples gave an ‘undetermined’ result (0.67%) and those tests were repeated, during which period the subjects were quarantined.

In ICON’s CRUs located in the US the percentage of samples found positive was 1.83% (53 out of 2891 tests performed) in the Salt Lake City (UT) location and 0.64% (15 of a total of 2330 tests) in the Lenexa (KS) site. The number of tests with an initially invalid result was in the order of 1%.

As a result of SARS-CoV-2 testing at check-in, in combination with other measures to reduce the risk, no known cases of transmission of the SARS-CoV-2 virus within any of the ICON’s CRUs exist.

**Figure 1.**
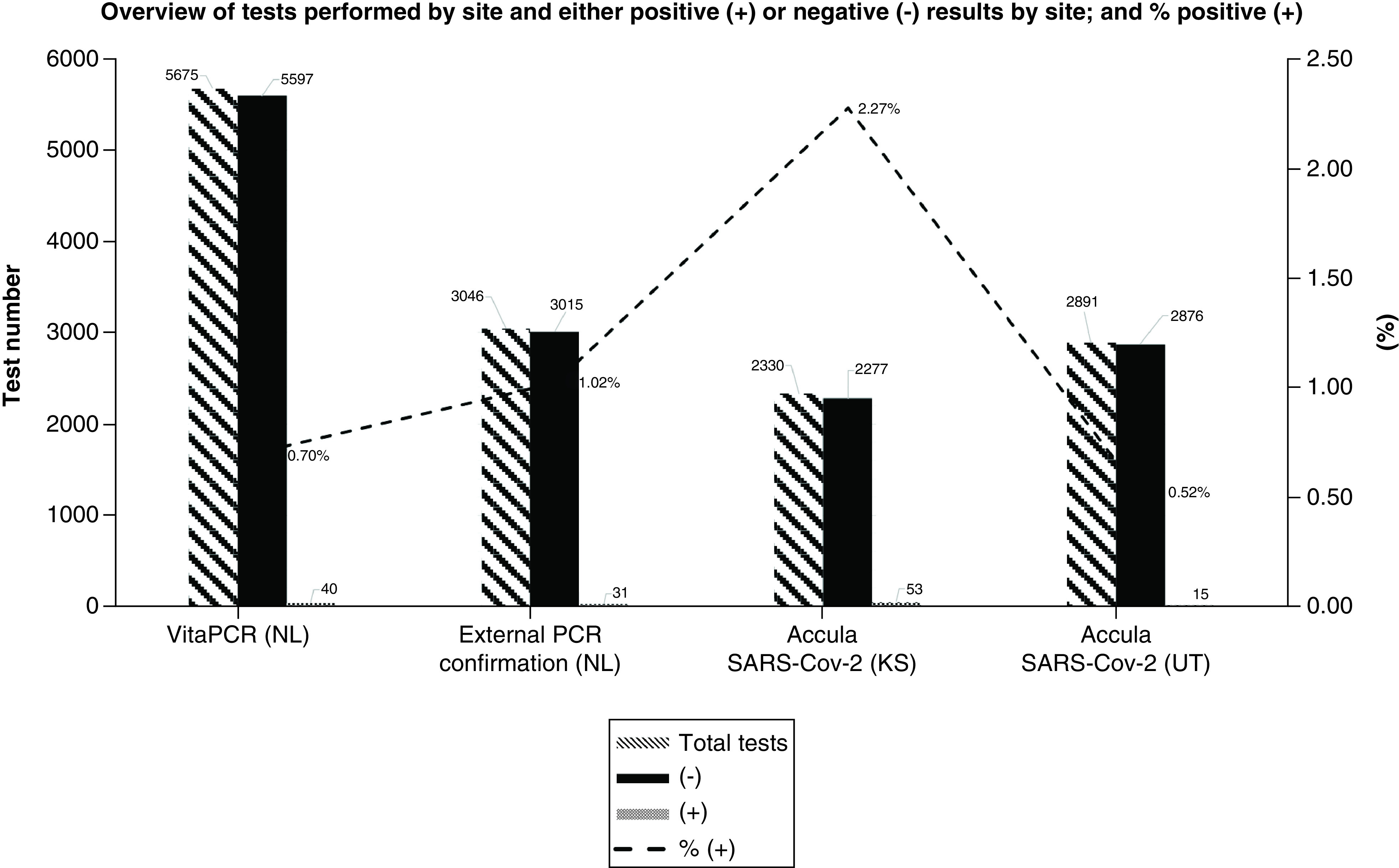
Number of tests performed at the different clinical research units. (-) denotes a negative test (no SARS-CoV-2 RNA detected above the threshold); (+) indicates a positive result. The dotted line shows the percentage of positive tests per test site.

Results from the PoC tests are shown in Figure 1.

In total 3046 samples from the NL CRU were sent to an external PCR testing facility, all from subjects participating in the clinical trials collected on or after day 2 of their residential period. The turnaround time of receiving the results was usually under 24 h for the Dutch situation. Of those samples, 31 were reported as positive (1.02%), two were undetermined (0.07%) and the remaining 98.92% were reported negative. In total ten of the 31 positive samples had Ct values <35, which is considered a clinically meaningful value indicating the potential to infect others [8]. Of all 31 positive samples, 13 had Ct values >35 and for the remaining eight samples no Ct value was reported.

For 13 study subjects, a positive result came back from the central PCR laboratory for the follow-up sample collected on day 2 or later of the residential period in the CRU. For some of these subjects, the positive result was then confirmed using another PoC PCR test, while for others the result could not be confirmed. Not for all subjects a confirmatory PoC test could be performed due to study and situation specific circumstances. Positive results that could not be confirmed were linked to high Ct values in the external PCR test. It was hypothesized that the sensitivity of the VitaPCR RT-PCR test performed as part of the screening procedures was limiting in these cases and that the subjects must have had remaining viral RNA from a cured prior infection. For the positive cases that were confirmed it was hypothesized that subjects were infected shortly before being screened at submission into the clinic and that they developed higher viral loads during the first days of their stay.

No data are available at this time for external confirmation tests conducted in ICON’s US CRUs.

## Discussion

During the early days of the pandemic, there were significant challenges in obtaining reliable data on the performance of various SARS-CoV-2 tests [9]. Data on assay sensitivity and specificity were limited and obtained from research on COVID-19 patients, often in a hospital setting. However, with the known likelihood that our testing would be primarily concentrated on asymptomatic healthy volunteers it was unclear whether testing these subjects for SARS-CoV-2 would add value to the general health check questionnaires (temperature monitoring, exposure, travel, etc.).

During the approximately 10 months since restarting the clinical trial activities in ICON’s CRU in the Netherlands, we identified 11 cases where asymptomatic volunteers tested positive at check-in, so the tests served their intended initial purpose of keeping infected asymptomatic subjects out of the clinics, with the exception of 13 subjects. Those subjects were identified using the centralized PCR test and may have been missed in the PoC screening test because of low viral loads from a prior infection or from infection right before screening assessments started. This shows that the application of the PoC test, together with the hygiene measures, personal protection, social distancing rules and responsiveness of the COVID-19 team, controlled the situation and avoided viral transmittance in the clinics. As far as we know, no infections from staff members or healthy volunteers alike within the CRU took place. In some cases, the PoC tests were considered less sensitive compared with the RT-PCR tests performed at external laboratories. Some of those cases were assigned to remaining viral RNA following a previous infection. In cases where a recent infection was missed at entry into the facilities, it is considered possible that also a central laboratory tests could not have detected that signal, but rather that after 2 days the viral load had increased. In those cases, the day 2 result from the external laboratory was also confirmed by the PoC test.

In the last months of 2020, antigen-based SARS-CoV-2 tests became commercially available on larger scales. We have also considered to replace the PCR based PoC test at check-in at ICON’s CRUs. The antigen-based tests are attractive given the rapid turnaround time of typically 15 min and their cost–effectiveness. However, due to the reduced sensitivity and accuracy of antigen-based tests, especially in asymptomatic persons and the need for confirmation testing with more accurate assays like RT-PCR based assays [10] it was elected to not deploy antigen-based tests at our facilities for admittance testing at check-in. We continue using the PoC PCR test as long as deemed necessary.

## Conclusion

In conclusion, the rapid PoC PCR-based tests, in combination with consistent safety practices such as face masks, social distancing, quarantining and risk-based exposure questions have shown to be reliable methods for continuing operations at CRUs and for safeguarding the safety of subjects participating in clinical trials and of the site staff. In addition, the PoC PCR tests have shown to be a useful tool for excluding SARS-CoV-2 infection in an asymptomatic healthy volunteer population. The results from our PoC tests in combination with gold standard PCR have been instrumental in keeping SARS-CoV-2 infections out of our clinics and thus limited the risk of COVID-19 on the conduct of clinical trials. Collaboration between experts from the bioanalytical laboratories, the clinical chemistry laboratories, the medical and operational staff in the CRUs and company management ensured safe and effective continuation of early phase clinical trials during the COVID-19 pandemic and helped to ensure safety of trial subjects.

## Future perspective

The COVID-19 pandemic had major implications on worldwide operations in many different sectors including clinical trial research. We anticipate that with the effect we have seen from the COVID-19 pandemic, the PoC strategy and other practices that we have taken to ensure safe continuity of CRU operations are valuable in preserving healthy volunteer safety and preventing a complete halt of clinical trial activities during the drug development phase, especially if we were to be faced with another devastating pandemic. With the importance that has been placed on drug development including the development of vaccines to fight these viruses, it is critical that we continue to learn from our current strategies to ensure that clinical trial research operates effectively and safely to allow for continuation of drug development. The future of the COVID-19 pandemic remains uncertain and; thus, the POC strategy and continued operation of Phase I clinical CRUs may be employed for years to come.

**Executive summary**

- The outbreak of the COVID-19 pandemic led to a disruption, including a slowdown or in some cases, halt, in many clinical research activities for the development of new drugs. Regardless of the devastation brought on by the SARS-CoV-2 virus, effective and continued operation of clinical research units conducting Phase I clinical trials remained an important step in the continuation of development of new drugs including vaccines to help fight the virus.
- A major challenge that was faced was developing a testing strategy for nonsymptomatic trial participants in an early stage of the outbreak.
- A point-of-care polymerase chain reaction test combined with a gold standard polymerase chain reaction test, strict social distancing and heightened hygiene measures allowed for limiting the number of infected subjects entering clinical research units and reduced further spread for the duration of the clinical trial.
- This strategy allowed for a safe and effective continuation of early phase clinical trials during the COVID-19 pandemic.
